# Structure-Guided Comparative Analysis of Proteins: Principles, Tools, and Applications for Predicting Function

**DOI:** 10.1371/journal.pcbi.1000151

**Published:** 2008-09-26

**Authors:** Raja Mazumder, Sona Vasudevan

**Affiliations:** Department of Biochemistry and Molecular & Cellular Biology, Georgetown University Medical Center, Washington, D.C., United States of America; Whitehead Institute, United States of America

## Introduction

With the increase in genomic and proteomic data from genome sequencing projects and structural genomic initiatives, we are faced with an increasing number of sequences and structures in various databases annotated as “uncharacterized,” “hypothetical,” or “unknown function” [1],[2]. In addition to this exponential increase in sequence and structure data, we are also seeing an increase in the number of databases that hold these data, and thus the need to evaluate the quality of these databases [3]. All these data, however, can be used meaningfully for biological and clinical research only if we can extract the functional information from them and convert biological data into knowledge of biological systems. While we have made significant progress in this regard with the availability of several functional prediction servers such as ProFunc, ProtFun 2.2, PFP ConFunc, and others [4]–[8], many challenges still remain in accurately inferring function and more importantly propagating this information reliably to the millions of proteins that still lack experimental characterization. Unfortunately, none of these servers have a high success rate for large-scale function predictions. The reasons for this failure are many-fold, including lack of strict adherence to common guidelines for functional inference. However, through rigorous and systematic comparative analysis of structures and sequences, one can make headway in annotating these proteins on a large scale with relevant biological functional information. Detailed methodologies for large-scale functional annotations are discussed elsewhere [9].

**Figure 1:**
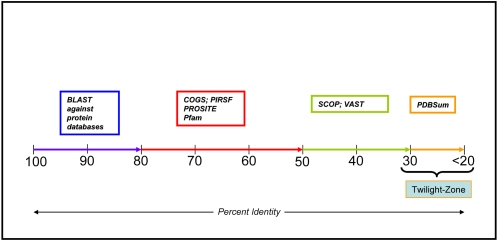
Percent-identity scale. The horizontal line gives the percent identity between query and subject sequences, and the boxes gives the resources and tools that can be used for functional inference.

Biological function can be inferred at different levels depending on sequence identities that exist between the sequences. The success of functional inference, however, depends on the availability of experimentally validated information of related proteins. This relatedness may be at the full-length protein level, domain level, structural level, or motif level. Depending on the type and level of similarity, specific or general functions can be propagated. In fact, it has become widely accepted that percent identity is more effective at quantifying functional conservation than any other scores or means [10]. Our view of this is presented as a percent-identity scale shown in Figure 1. This scale is rather conservative since it is not clear what level of sequence identities guarantees that two proteins have similar functions [11],[12]. For sequences with identities above 50%, a general approach for functional characterization is by transfer of annotation from a characterized template to a subject. While it is a common practice to transfer such annotations, an error rate as high as 30% or more has been reported when proper caution is not taken [13]. Therefore, ideally for sequences whose identities fall below this threshold, availability of structural information becomes important, and transfer of annotation should be done with care. An example where homology-based transfer failed is cbiT, which was annotated as a decarboxylase until the structure revealed that it was a methyltransferase [14]. It has now become clear from several studies that no single method is sufficient for functional inference [15],[16]. In fact, as will be clear from the example discussed in this tutorial, several layers of evidence have to be collected before assigning the function to a protein.

**Figure 2:**
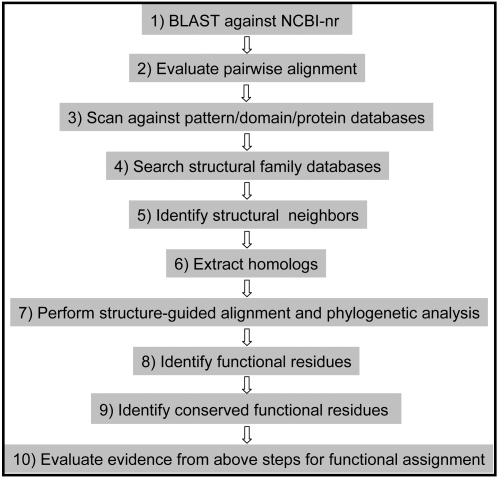
Ten-step procedure for comparative analysis of protein structures and sequences to infer biological function.

The main objective of this article is to define a ten-step procedure (Figure 2) guided by the percent-identity scale (Figure 1), that can be followed as a general rule for functional inference of an uncharacterized protein. In addition, the goal is also to provide the available tools and databases that are relevant for functional analysis.

We will describe the ten-step procedure using an example of an uncharacterized conserved bacterial protein from *Aquifex aeolicus* (UniProt ID O67940_ *AQUAE*) [17]. Aquifex, a hyperthermophilic chemolithoautotrophic bacterium, is considered to be one of the earliest bacteria to diverge from eubacteria [18]—hence its importance. Also, bacterial halogenation is poorly understood, and this example brings out the importance and challenges in function prediction.

### 

#### *Note*

*The analysis performed and results shown reflect the databases at the time of writing of this paper. Unless otherwise mentioned, default parameters were used. Also, because of limitation in space, we have not included other excellent databases and tools that can be used for this type of analysis. The list of tools and resources included in this paper (Table 1) were chosen because of the authors' familiarity with them, and because they are widely used.*

**Table 1:** URLs used for this tutorial

Resource	URL
UniProt	http://www.uniprot.org
NCBI	http://www.ncbi.nlm.nih.gov
PDB	http://www.pdb.org
SCOP	http://scop.mrc-lmb.cam.ac.uk/scop/
PIRSF	http://pir.georgetown.edu/pirsf/
COGs/KOGs	http://www.ncbi.nlm.nih.gov/COG/
PROSITE	http://expasy.org/prosite/
VAST	http://www.ncbi.nlm.nih.gov/Structure/VAST/vast.shtml
Cn3D/CDTree	http://www.ncbi.nlm.nih.gov/Structure/cdtree/cdtree.shtml
PDBSum	http://www.ebi.ac.uk/thornton-srv/databases/pdbsum/

## Tools, Resources, and General Concepts for Functional Analysis and Annotation Transfer

### 

#### (a) Homology determination based on full-length sequence information

Based on the percent-identity scale (Figure 1) for sequences with identities >80%, a simple pair-wise alignment or comparison using BLAST [19] to an experimentally characterized protein may suffice to infer function, provided the uncharacterized protein and the characterized protein are of similar lengths and align end-to-end without large insertions or deletions. In such cases, for the most part it may be safe to assume that the two proteins have similar overall functions. The widely used and the most reliable resource for obtaining high-quality annotated sequences is UniProtKB/Swiss-Prot [17]. For sequences whose identities fall in the 50%–80% range, the general approach for functional assignment includes evaluation of homology to protein family, domain, and functional motif databases. The most commonly used methodology is querying against profiles generated using either hidden Markov models (HMM) [20] or position-specific scoring matrices (PSSM) [19].

In the higher end of this range, say above 70% identity, a widely used practice is to see if the query protein belongs to a protein family that has experimentally characterized members. The concept of protein family based on homology was articulated by Margaret Dayhoff in the early days of sequence analysis [21]. Protein family classification has several advantages as a basic approach for large-scale genomic annotation over other methods. Classification databases ideal for this kind of analysis include PIRSF [22] and the prokaryotic and eukaryotic Clusters of Orthologous Groups of proteins (COGs and KOGs) [23],[24]. The PIRSF provides classification of UniProtKB sequences primarily into homeomorphic (end-to-end similarity) families and subfamilies (domain level superfamilies are also included) based on their evolutionary relationships. Because PIRSF families and subfamilies are based on full-length proteins rather than on component domains, they allow annotation of generic biochemical and specific biological functions, as well as classification of proteins without well-defined domains. On the other hand, COGs and KOGs consist of clusters of orthologous (and co-orthologous/inparalogous) proteins from completed genomes. The identification of orthologous protein sets is based on automatic clustering of proteins from three or more distantly related organisms based on reciprocal BLAST. This is followed by additional automatic recruitment based on a rigorous BLAST-based algorithm, and subsequent extensive manual curation of membership (including splitting of full-length proteins and assigning them to different clusters if necessary) and annotation.

For sequences whose identities fall in the lower end, say <70% range, in the absence of end-to-end similarity, a safer approach would be to evaluate domain architectures of these proteins, as these can evolve and exist independently of the rest of the protein chain. The most widely used domain database that provides a comprehensive coverage is Pfam [25].

#### (b) Homology determination based on 3D-structural information

Sequence similarity based on full-length sequences has been used as a guiding principle in many classification databases. While this works quite well for closely related sequences whose sequence identities are greater than 50%, it begins to fail for sequences that are related at the three-dimensional structural levels rather than at sequence levels [1], [26]–[28]. This is not surprising since molecular evolution conserves structural features longer than sequence [16],[29].

Examination of a protein's structural neighbors and fold comparisons can reveal distant evolutionary relationships that are otherwise undetectable and, perhaps, suggest unsuspected functional properties. Just as proteins with end-to-end similarities may be evolutionarily related, structures with similar folds may also be related. Data resources that provide structural comparisons include Vector Alignment Structural Tool (VAST) [30], Combinatorial Extension (CE) [31], and DALI databases [32]. For structural classifications, SCOP and CATH have become the most widely used structural resources that provide a comprehensive hierarchical description of structural relationships [33]–[35]. The uniqueness of SCOP, however, is that it is an expert-constructed database geared toward identifying evolutionary relationships rather than relationships based on mere three-dimensional geometry of proteins.

#### (c) Sequence and structural motifs to aid in functional inference

Analysis of sequence/structural motifs becomes valuable especially for cases where the overall percent identity goes below 30% for functional inference. These functional motifs/sites form stable units and are evolutionarily conserved relative to the remainder of the protein. Their identification is important in the assignment of protein names and accurate propagation of structural and functional site annotations [9]. The most commonly used programs and tools available to calculate inter and molecular contacts are PDBSum [36] and LPC/CSU [37] servers. For identifying known sequence and structural patterns/motifs, PROSITE and the Catalytic Site Atlas (CATRES), respectively, are invaluable resources [38],[39].

## Ten-Step Procedure—An Example

We propose a ten-step procedure (Figure 2) that can generally be followed for inferring function of an unknown protein. The candidate protein with ID O67940_*AQUAE* from *Aquifex aeolicus* is currently annotated as an “*uncharacterized conserved protein*” in UniProtKB [17], whose orthologs are found in bacterial and archeal species.

### 

#### Step 1: PSI-BLAST against NCBI non-redundant database (nr)

**Figure 3:**
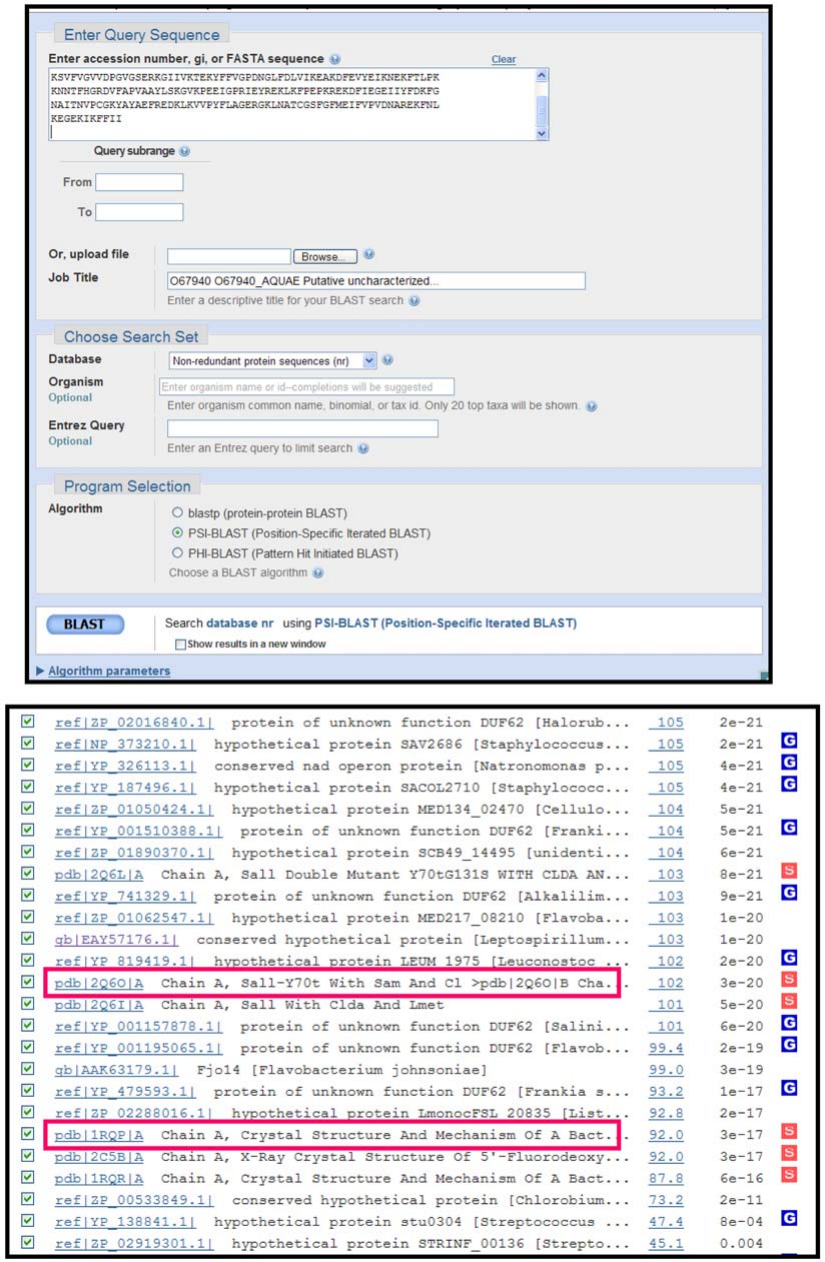
PSI-BLAST input panel (top) and PSI-BLAST output iteration (bottom). (Top) Default parameters are used. The fasta sequence of query protein with UniProt accession O67940 from *Aquifex aeolicus* is blasted against NCBI's nr database. (Bottom) The query protein *O67940_ AQUAE* hits several structures (tagged with S in a red box). Only two of the non-redundant structures with PDB-ids 2Q6O and 1RQP (marked by a pink box) are functionally characterized with e-values 3e-20 and 3e-17 and percent identities of 32% and 26%, respectively. (The Expect value (E) or an e-value is a parameter that describes the number of hits one can “expect” to see by chance when searching a database of a particular size. It decreases exponentially as the Score (S) of the match increases.)

The amino acid sequence of O67940_ *AQUAE* is blasted against NCBI's non-redundant protein database (nr) in order to retrieve all its related sequences (Figure 3, top). Results of the BLAST output (Figure 3, bottom) show no hit to a characterized protein among the top hits (additional iterations to convergence did not hit any other characterized members). However, a close examination of the results indicates that the query protein hits several solved crystal structures (tagged with S in a red box). Two of them with PDB IDs 2Q6O from *Salinispora tropica* (UniProt accession A4X3Q0) and 1RQP from *Streptomyces cattleya* (UniProt accession Q70GK9) are functionally characterized as chlorinase and fluorinase, respectively [40]–[42]. In the BLAST results, 2Q6O has an e-value of 3e-20 with a percent identity of 32%, while 1RQP has an e-value of 3e-17 with a percent identity of 26%. Now the question is: Can we reliably predict O67940_Aquefix to be a chlorinase (specific to a chloride ion) or a fluorinase (specific to a fluoride ion) or just a halogenase (could be specific to one or more of the halogens)? The answer is not yet known since the sequence identities between the query and the characterized members fall in the low end of the sequence-identity scale, and therefore additional supportive evidence needs to be gathered before reliable function transfer.

#### Step 2: Evaluate pairwise alignment with the identified structures from Step 1

**Figure 4:**
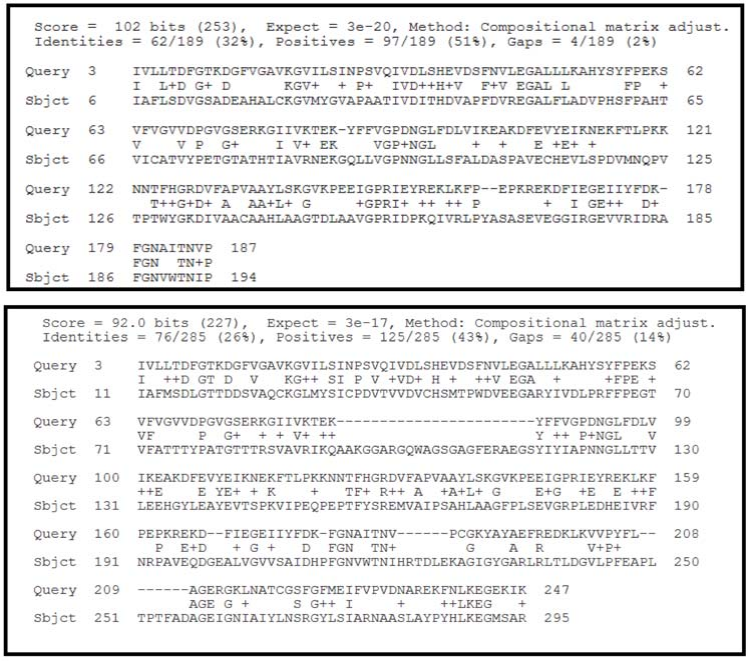
Pairwise alignment between query sequence *O67940_ AQUAE* and 2Q6O (top) and 1RQP (bottom). (Top) Query aligns end-to-end without any long gaps with a sequence identity of 32%. (Bottom) Query aligns end-to-end but with three regions of gaps, the most significant being a 23-residue region in 1RQP residues 92–116. The sequence identity of query with 1RQP is 26%.

The results of the BLAST run (Figure 4) of query versus subjects (2Q6O—pdb|2Q6O|A and 1RQP—pdb|1RQP|A) gives us the pairwise alignments. The pairwise alignment of query with 2Q6O (Figure 4, top) extends almost the entire length of the protein without long gaps. However, the alignment of query with 1RQP (Figure 4, bottom) has three regions with relatively long gaps. Based on this, it is clear that we need to get additional homologs and construct a multiple sequence alignment to identify the conserved residues before transferring functional annotation.

#### Step 3: Scan against sequence pattern, domain, and family classification databases

**Figure 5:**
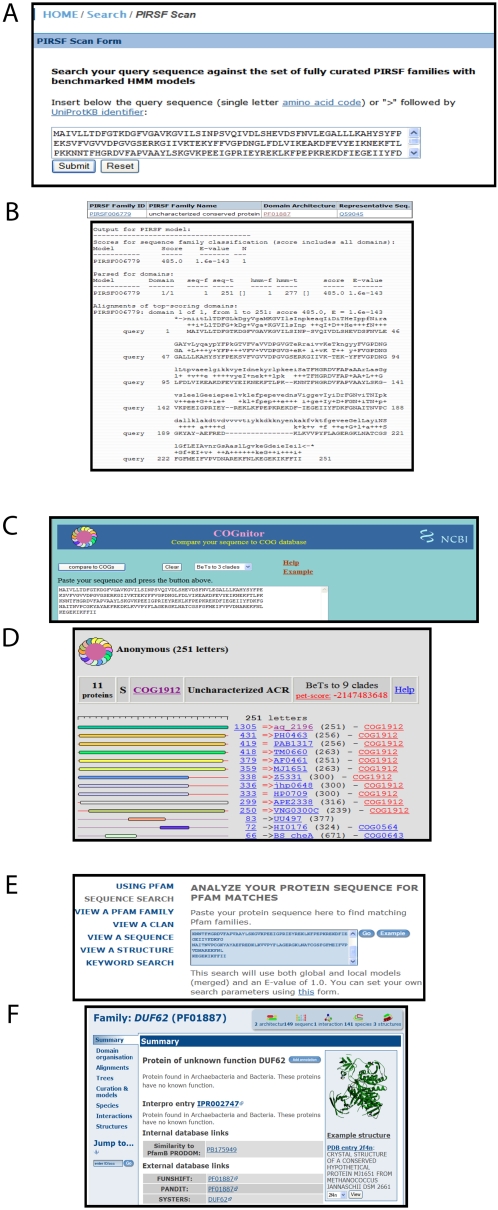
PIRSF (A,B), COG (C,D), and Pfam (E,F) input and results. (A) The fasta sequence of query protein with UniProt accession O67940 from *Aquifex aeolicus* is scanned against PIR's curated family database. (The query is searched against the full-length and domain hidden Markov models for manually curated PIRSFs. If a match is found, the matched regions and statistics are displayed). (B) The query hits the PIRSF family PIRSF006779. The output provides family details; statistical data for full-length proteins, composite domains, and a pairwise alignment of query with the consensus sequence of the PIRSF. (C) The fasta sequence of query protein with UniProt accession O67940 from *Aquifex aeolicus* is scanned against the database of clusters of orthologous groups. COG compares protein sequences encoded in complete genomes, representing major phylogenetic lineages. Each COG consists of orthologous/co-orthologous proteins from at least three lineages. (D) The query hits COG1912. The output provides the family details: statistical score, reciprocal best hits, and members of the family. (E) The fasta sequence of query protein with UniProt accession O67940 from *Aquifex aeolicus* is scanned against the Pfam domain database. The Pfam database is a large collection of domain families, each represented by multiple sequence alignments and hidden Markov models (HMMs). (F) The query hits Pfam family PF01887.

Results obtained from the steps so far are not conclusive to determine if the query is a chlorinase or a fluorinase. In this step, we will attempt to see if the query protein belongs to any well-annotated protein and domain families or if the protein has any specific identifiable sequence pattern. The results of scanning the candidate protein against family databases PIRSF and COGS are given in Figure 5. The query along with 2Q6O and 1RQP belong to PIRSF006779 and COG1912; both families, however, lack any functional annotation. Similarly, scanning against the domain database Pfam (Figure 5E and Figure 5F) and functional site database PROSITE does not provide any additional insights into the function of the query protein O67940_AQUAE. Nevertheless, Steps 1, 2, and 3 provide clues about phyletic distributions of homologs that can be used to construct a multiple sequence alignment.

#### Step 4: Search against structural family databases for structural classification

**Figure 6:**
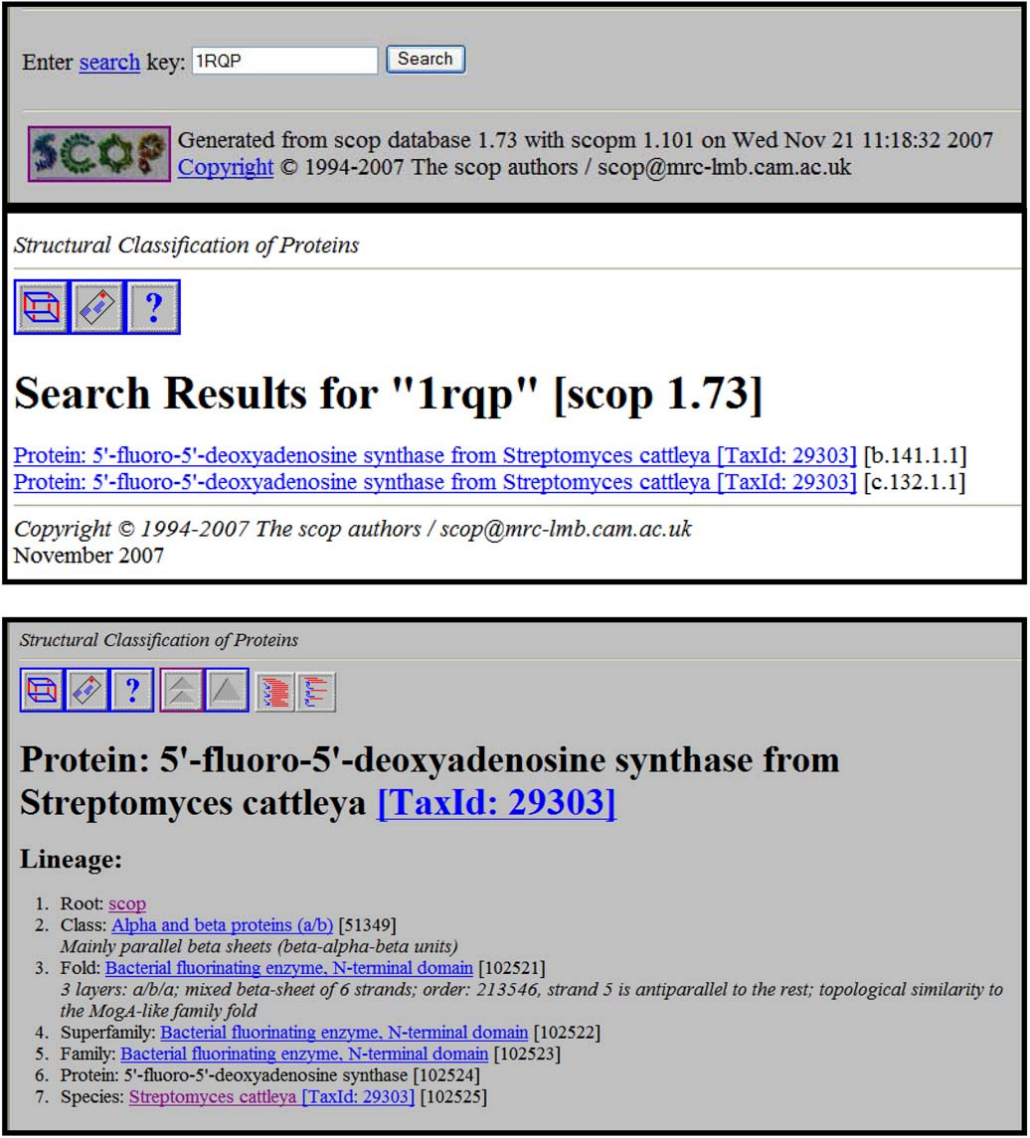
SCOP output. 1RQP is used since our query protein O67940 from *Aquifex aeolicus* does not have a solved structure. The results indicate that the N-terminal and C-terminal domains of 1RQP belong to two SCOP superfamilies. (The SCOP database provides a detailed and comprehensive description of the structural and evolutionary relationships between all proteins whose structure is known).

Similarity between related sequences at either the sequence or structural levels may give important clues about their functions since it may be a consequence of functional or evolutionary relationships. Results of the structural searches using the SCOP database is presented in Figure 6. The results indicate that the N- and C-terminal domains of 1RQP belong to two SCOP superfamilies named Bacterial fluorinating enzyme (N-terminal domain) and Bacterial fluorinating enzyme (C-terminal domain). 2Q6O is not classified in the SCOP 1.73 release, but most likely belongs to the same superfamily as 1RQP.

#### Step 5: Search structural database for structural neighbors

**Figure 7:**
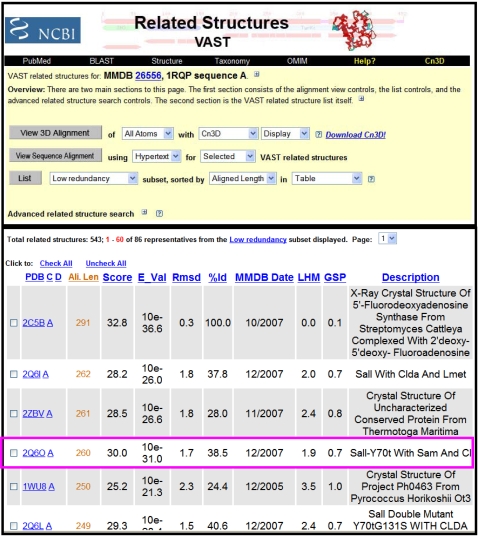
VAST output. Since our query protein O67940 from *Aquifex aeolicus* does not have a solved structure, 1RQP is used as a query. The only non-redundant structural neighbor that provides functional annotation is 2Q6O, indicated by a pink box.

This becomes an important step especially for sequences whose percent identity falls below 30%. Since our query does not have a structure, 2Q6O and 1RQP will be used as starting points to get other related structures. Results of the structural searches using VAST is presented in Figure 7. Thus, identified structures can be used to generate a high-quality structure-guided multiple sequence alignment to which the query and other related sequences can be aligned. The generation of a high-quality alignment is critical for function prediction and reliable phylogenetic analysis.

#### Step 6: Extract homologs

Transfer of annotations from one homolog to another is not always straightforward. To transfer annotation, one has to identify homologs that can be used for constructing multiple sequence alignments and subsequently used for performing phylogenetic analysis to identify orthologs (next step). More often than not, when many paralogs are present, it becomes difficult to identify a true ortholog. This step is to identify homologs based on results obtained from earlier steps. With the increasing number of genomes being sequenced, it is becoming apparent that restricting analysis to high-quality genomes and sequences from model organisms for generating alignments and performing phylogenetic analysis is important.

#### Step 7: Perform structure-guided alignment and phylogenetic analysis

**Figure 8:**
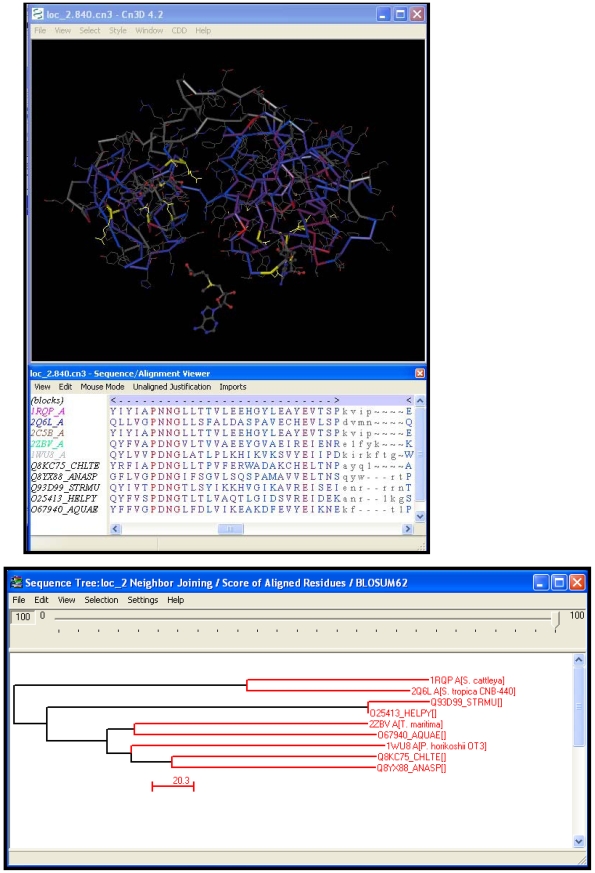
Structure-guided alignment constructed with homologous sequences using Cn3D (top) and neighbor-joining tree based on the score of aligned residues from homologous sequences using CDTree (bottom).

High-quality multiple alignments are a pre-requisite for understanding the evolutionary relationships that exist between homologous sequences. A structure-guided alignment carried out using Cn3D on the structures and sequences obtained from Step 6 is presented in Figure 8. This alignment is manually edited to ensure that all the secondary structural elements are properly aligned without any geometric violations. To this manually edited structural alignment, the initial query O67940_Aquefix along with the identified homologs from Step 6 are added. It is interesting to note that the longest gap observed in the BLAST pairwise alignment in Step 1 (Figure 4, bottom) between query and 1RQP corresponds to an exposed loop region of the protein. This 23-residue loop region absent in both 2Q6O and the query seems to be significant enough to cause a decrease in the buried surface area around the active site compared to 1RQP. Neighbor-joining (NJ) phylogenetic analysis of the aligned sequences was carried out using CDTree. The tree reveals that the query and our subjects (1RQP and 2Q6O) do not fall in the same branch (Figure 8, bottom). This indicates that transfer of annotation requires more in-depth analysis that includes examination of structural attributes such as regions around the active and binding sites. As mentioned earlier, conservation of these sites is critical for functional inference.

#### Step 8: Identify functional residues

**Figure 9:**
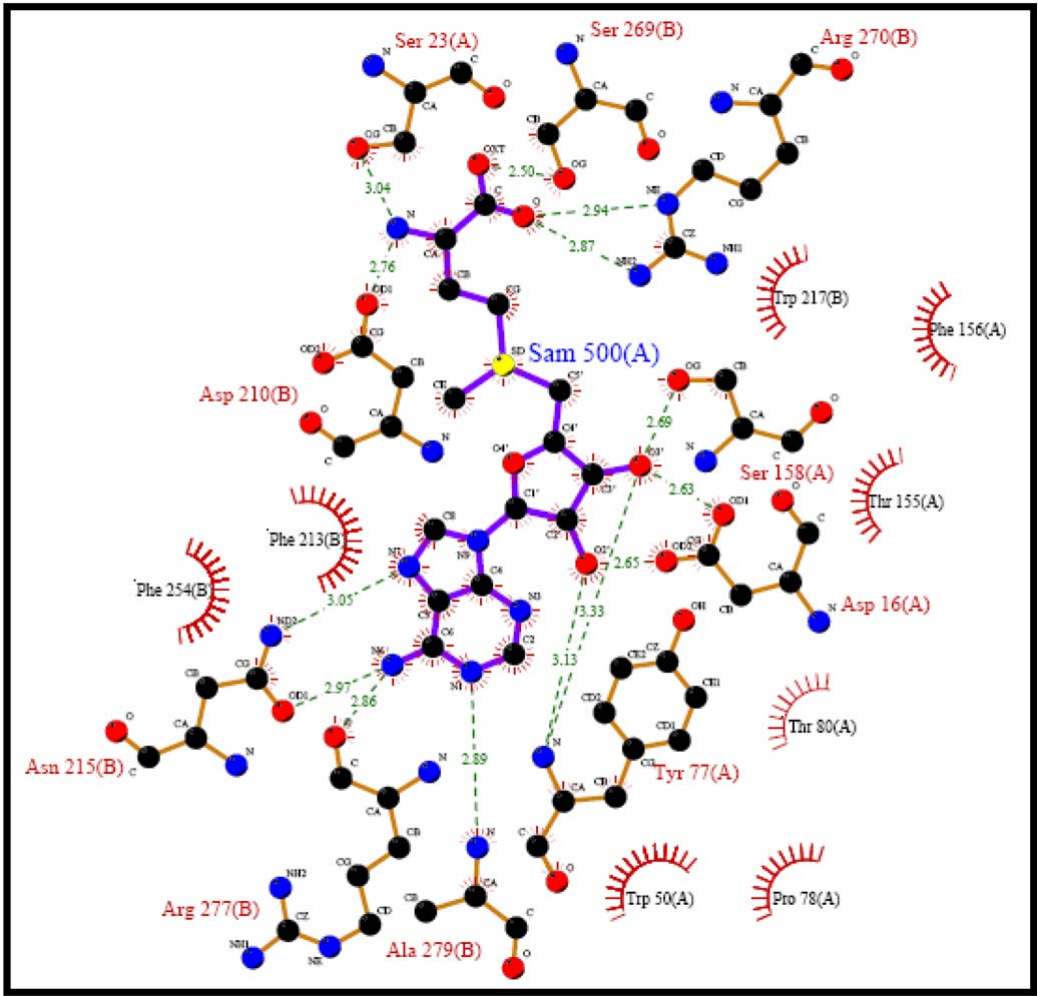
Ligplot for 1RQP. SAM-binding residues. Dashed green lines indicate hydrogen bonds, and the half-moon indicates van der Waals interactions. (Ligplot is a program for automatically plotting protein–ligand interactions provided as part of the PDBsum database, which is a Web-based database of summaries and analyses of all PDB structures).

Structures of complexes provide more functional information than uncomplexed structures. 2Q6O, also referred to as SalL, is a trimer with substrate chloride and ligand S-adenosyl-L-methionine (SAM) bound. 1RQP on the other hand is a hexamer (dimer of trimers) with three molecules of the ligand SAM bound. The functional site in these two related structures reside at the interface between the monomers. SAM-binding residues were obtained from PDBSum [36]. A plot of SAM-binding residues for 1RQP is shown in Figure 9. 2Q6O is a SAM-dependent chlorinase that catalyzes the transfer of a chloride ion to SAM to generate 5′-chloro-5′-deoxyadenosine [41]. It has also been shown to possess brominating and iodinating activities but not fluorinating activity. 1RQP on the other hand is a fluorinating enzyme that catalyzes the formation of a C–F bond by combining SAM and F^−^ to generate 5′-fluoro-5′-deoxyadenosine and L-methionine [43]. Subsequently, it was shown that fluorinase from *Streptomyces cattleya* is also a chlorinase [44]. There are a few crucial differences between 1RQP and 2Q6O that give them their halogenating specificities. For example, the active site residue (involved in catalysis) Gly 131 in 2Q6O is Ser 158 in 1RQP. This small difference seems to result in a larger binding pocket in 2Q6O, resulting in the apparent differences in their specificities, making one a fluorinase/chlorinase and the other a chlorinase/brominase/iodinase. In addition, mutagenesis studies indicate another important active site residue Thr 70 in 1RQP, occupied by a hydrophobic residue Tyr 70 in 2Q6O. Mutation of Tyr 70 in 2Q6O to Thr decreases the chlorinating and brominating activities, indicating their important role in catalysis and the observed specificities [41].

#### Step 9: Identify conserved functional residues in query

Mapping the functional residues from 1RQP and 2Q6O (Table 2) to query O67940_ AQUAE identifies residues Asp∶8, Phe 15, *Val 67, Asp 69, *Gly 127, Asp 177, Asn 181, Ser 221, Phe 222, Leu 229, and Val 231 as part of the catalytic region. The two crucial active site residues (marked with a *) discussed in the previous step, namely Gly 131 and Tyr 70 (mutated to Thr) in 2Q6O, are Gly 127 and Val 67 in the query. Alignment of homologous sequences carried out in Step 7 indicates that this position is occupied predominantly by a hydrophobic residue, except in the case of the fluorinating enzyme 1RQP where it is a Thr.

**Table 2:** Alignment of functional residues

ID/Acc	Functional residues (binding and catalytic sites)										
1RQP	Asp 16	Ser 23	*Thr 75	Tyr 77	*Ser 158	Asp 210	Asn 215	Ser 269	Arg 270	Arg 277	Ala 279
2Q6O	Asp 11	Ala 18	* +Tyr 70	Tyr 72	*Gly 131	Asp 183	Asn 188	Ser 242	Arg 243	Arg 250	Glu 252
O67940	Asp 8	Phe 15	*Val 67	Asp 69	*Gly 127	Asp 177	Asn 181	Ser 221	Phe 222	Leu 229	Val 231

*: indicates catalytic sites.

**+:** Tyr70Thr mutation in 2Q6O.

#### Step 10: Evidence-based assignment of biological function of query O67940_Aquefix

Based on the conservation of the crucial residues that are involved in catalysis, the query is closer to the chlorinating enzyme 2Q6O than the fluorinating enzyme 1RQP. While it is safe to assume that the binding site for SAM is conserved among the members of PIRSF006779 and that all its members bind to SAM and likely are halogenases, it is not safe to assume that all the members are chlorinases or fluorinases. Their specificities may be to a fluoride, chloride, bromide, or iodide. Judging from the alignment and available experimental evidence on bacterial fluorinating (and chlorinating) enzymes in *Streptomyces cattleya* [45],[46] and chlorinating enzyme from *Salinispora tropica*, it is likely that the query protein O67940_Aquefix is an enzyme that can halogenate SAM with chloride, bromide, or iodide ions. Based on available experimental information, it is not possible to say if the *Aquefix* enzyme can also use fluorine. Additional supporting experimental data need to be collected before we can conclude the precise specificity of the query.

By following all the above steps, we have answered one critical question that we set out to answer at the beginning of this tutorial, i.e. the function of O67940_ *AQUAE*. In addition, we have also identified functional residues.

## Summary

The main objective of this article was to define a ten-step procedure, largely guided by the percent-identity scale, that can be followed as a general rule for functional inference of an uncharacterized protein. This procedure is by no means exhaustive but can be used as an initial process for functional assignment. In many cases, additional clues and complementary information may be obtained from pathway analysis, operon information, and other non-homology based methods. We have demonstrated how by following the ten steps a function could be assigned for an uncharacterized conserved protein with its related sequences. In addition, the goal was to provide an overview of the available tools and databases to carry out comparative sequence and structural analysis.
